# Assessment of echinococcosis control in Tibet Autonomous Region, China

**DOI:** 10.1186/s40249-022-00987-9

**Published:** 2022-05-26

**Authors:** Liying Wang, Quzhen Gongsang, Huasheng Pang, Min Qin, Ying Wang, Jingzhong Li, Roger Frutos, Laurent Gavotte

**Affiliations:** 1 National Institute of Parasitic Diseases, Chinese Centre for Disease Control and Prevention (Chinese Centre for Tropical Diseases Research), NHC Key Laboratory of Parasite and Vector Biology, WHO Collaborating Centre for Tropical Diseases, National Centre for International Research On Tropical Diseases, Shanghai, 200025 China; 2 Tibet Center for Disease Control and Prevention, NHC Key Laboratory of Echinococcosis Prevention and Control, Lhasa, 850000 China; 3 Cirad, UMR 17, Intertryp, Campus international de Baillarguet, 34398 Montpellier, France; 4 Espace-Dev, UMR D-228, Université de Montpellier, 34000 Montpellier, France

**Keywords:** Echinococcosis, Hydatidosis, Source of infection, Control measure, Effect assessment, China

## Abstract

**Background:**

In China the highest prevalence of echinococcosis is in Tibet Autonomous Region (TAR). The government has issued documents and implemented comprehensive prevention and control measures focusing on controlling the source of infection of echinococcosis. It was very important to understand the implementation and effect of infectious source control measures. The purpose of this study was to examine the implementation of measures to control infectious source (domestic and stray dogs) in TAR and to assess their effectiveness.

**Methods:**

We collected data on domestic dog registration and deworming and stray dog sheltering in 74 counties/districts in the TAR from 2017 to 2019. Fecal samples from domestic dogs were collected from randomly selected towns to determine *Echinococcus* infection in dogs using coproantigen ELISA. We analyzed the data to compare the canine rate of infection between 2016 and 2019. The data analysis was performed by SPSS statistical to compare dog infection rate in 2016 and 2019 by chi-square test, and ArcGIS was used for mapping.

**Results:**

From 2017 to 2019, 84 stray dog shelters were built in TAR, and accumulatively 446,660 stray or infected dogs were arrested, sheltered, or disposed of. The number of domestic dogs went downward, with an increased registration management rate of 78.4% (2017), 88.8% (2018), and 99.0% (2019). Dogs were dewormed 5 times in 2017, 12 times in 2018, and 12 times in 2019. The dog infection rate was 1.7% (252/14,584) in 2019, significantly lower than 7.3% (552/7564) from the survey of echinococcosis prevalence in Tibet in 2016 (*P* < 0.05).

**Conclusion:**

Between 2017 and 2019, the number of stray dogs and infection rate of *Echinococcus* spp. in domestic dogs decreased significantly, indicating that dogs were effectively controlled as a source of infection in TAR and reflecting a significant decrease in the risk of echinococcosis transmission.

**Graphical Abstract:**

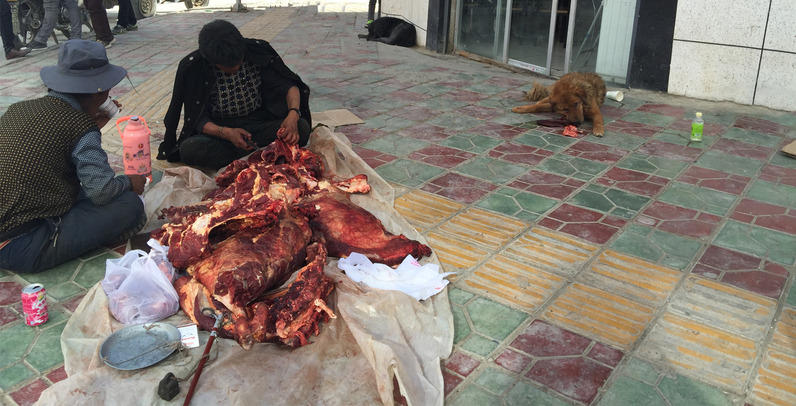

## Background

Echinococcosis, which is a zoonotic parasitic disease, caused by the larvae of *Echinococcus.* In China, two major types of echinococcosis are prevalent: cystic echinococcosis (CE) which is caused by the larvae of *E. granulosus* and alveolar echinococcosis (AE) which is caused by the larvae of *E. multilocularis* [1]. It is estimated that there are at least 188,000 new CE cases worldwide every year, resulting in 1,097,000 disability-adjusted life years (DALYs), and China accounts for 40% of the world [2]. The disease burden of AE is 666,434 DALYs per year in the world, and 91% of cases and 95% of DALYs occur in China every year [3]. Echinococcosis is highly endemic in western and northern China. Tibet Autonomous Region (TAR), also simply referred to as Tibet, located in the Qinghai-Tibet Plateau, which is one of the most infected regions. A national survey of echinococcosis, which conducted between 2012 and 2016, showed that echinococcosis prevalence in humans was 1.66% and estimated that nearly 50,000 patients experienced echinococcosis [4]. In the survey, the prevalence of echinococcosis was found to be highest in TAR [4]. Echinococcosis prevailed in all 74 counties of TAR. In animals, the disease prevalence was 7.30 and 13.21%, in dogs and livestock respectively [5]. As definitive hosts, dogs may significantly impact transmission and dissemination of *E. granulosus* [6]. Dogs, together with small mammals, are also involved in the transmission of *E. multilocularis* [7]. When dogs prey on small mammals, they can carry *E. multilocularis* into a synanthropic transmission ecosystem [8]. In addition, domestic dogs are identified as the most important definitive host of both *E. granulosus* and *E. multilocularis* with the highest risk of transmitting CE and AE to humans due to their ability to wander freely in pastoral areas and prey on slaughtered livestock [9]. Few people pass scores from echinococcosis prevention questionnaire [10]. These factors make TAR the region with the most severe prevalence worldwide. Echinococcosis has become a major public health issue that seriously restricts economic development, ethnic unity and social stability of TAR, and seriously endangers health and safety of people. It is also a major obstacle to the development of TAR [11].

In March 2017, in response to the severe echinococcosis situation, the General Office of the People’s Government of the Tibet Autonomous Region issued the “Work Plan for Comprehensive Control of Echinococcosis in the Tibet Autonomous Region (2017–2020) (ZZBF [2017] No. 29)”, hereinafter referred to as “the Plan”, to speed up and strengthen echinococcosis control in TAR. The Plan requires complete screening on all populations and implementation of various control measures (focusing on infection source control). This work was conducted in order to assess the implementation of measures to control infectious source (domestic dogs and stray dogs) for echinococcosis in TAR. Our findings will provide reference for the implementation of further prevention and control measures.

## Methods

### Study area

TAR is located in the southwest of Qinghai Tibet Plateau, with an average altitude of more than 4000 m. TAR has 74 endemic counties (districts) in 6 prefecture-level cities (Lhasa City, Changdu city, Shannan city, Shigatse city, Naqu city, Linzhi city) and one prefecture (Ali Prefecture). Among these counties 47counties are endemic counties for mixed CE and AE, and all the 74 counties are endemic counties for CE.

### Source of data

The Center for Disease Control and Prevention of the Tibet Autonomous Region (TAR CDC), guided by the National Institute of Parasitic Diseases (NIPD), Chinese Center for Disease Control and Prevention (China CDC), prepared a questionnaire for identifying the infection sources − dogs from 2017 to 2019. Data on dog infection rates of *Echinococcus* spp. were collected from endemic counties in 2019 in conjunction with the Annual Task of Central Government's Transfer Payment Project for Echinococcosis Control.

### Implementation of control measures

The TAR government made the prevention and control of echinococcosis a priority and thus incorporated echinococcosis control into government's performance evaluation. Leadership groups for echinococcosis control have been established at provincial, regional, and county levels. Comprehensive measures were implemented to prevent and control echinococcosis in all 74 endemic counties (districts) in six prefecture-level cities and one prefecture in TAR. In endemic counties, relevant departments actively functioned according to requirements of prevention and control programs, implemented comprehensive control strategies and measures focusing on infection source control and promoted dog registration and management. To ensure effective dog management, the public security bureau of each county of each city (prefecture) established a leadership group for dog management according to specific requirements from the Opinions on Regulating Dog Management in TAR and the Plan. Based on dog statistics, township (town) police stations, convenient police posts, and village resident policemen implemented dog management requirements, such as restriction, tethering and permit application, into each household in each village. The public security department collaborated with agriculture and animal husbandry department to collect and record basic dog information. Registered dog owners and dogs were photographed and put on record for dynamic and standardized management. A proactive campaign was launched to educate farmers and herders about the importance of limiting the number of dogs and tethering dogs. Currently, all dogs in cities (prefectures) are tethered. For many stray dogs, the measures focused on territorial centralized accommodation and management and disposal of infected dogs. The situation in the Linzhi Prefecture has previously been studied by Wang et al. [12] and data were added to the results of this study.

### Dog registration and management

According to the “Opinions on Regulating Dog Management in TAR”, each household should have no more than two dogs, and these dogs should be tethered. Shepherd dogs should also be tethered immediately upon arrival at the temporary settlement, and dog feces should be buried. Dog deworming registration cards were established to register all domestic (herd) dogs in endemic areas and register ownerless dogs by village. Contents of the registration were: name of the head of household, dog’s sex, age, fur color, and date of each deworming. These tasks were completed by local public security departments and agricultural departments.

### Dog deworming

Efforts have been made to ensure that each dog is dewormed monthly following relevant requirements from the Technical Plan for Echinococcosis Control (2008 Edition) issued by the Central Government. The local agriculture and husbandry department sent down deworming drugs and provided instructions on conducting operations. Dog owners embedded praziquantel into food such as zanba to feed dogs and recorded it on a logbook every month. Praziquantel (specification: 0.2 g/tablet) was used to deworm all dogs, at 1 to 2 tablets/dose/dog (2 tablets for dogs > 15 kg). The dose was delivered once a month. Dogs were fed with food-coated drugs. The drug was ensured to be swallowed and the treatment was recorded on a dog deworming registration card.

### Disposal of dog feces after deworming

Dog feces were collected and disposed (buried in-depth or incinerated) within five days after deworming to prevent *Echinococcus* eggs from contaminating the environment.

### Reduction of dog populations

Various measures were taken to control the number of dogs, stray dogs were accommodated where conditions allow, and infected dogs were hunted down. The public security bureau of each county (district), under unified instructions of comprehensive echinococcosis control leadership group at each level, invited third-party capture teams to cooperate with public security and armed police to make joint efforts in stray dog capture and sheltering. Farmers and herders were informed about the restrictions on dog breeding. Number of domestic and stray dogs collected by local veterinarian.

### Monitoring of dog infection

One administrative village in each endemic township was randomly selected each year. According to dog deworming registration cards, 20 households in the village were identified using a systematic random sampling method. One sample of feces from each household was collected to obtain 20 samples. Whenever the number of samples was less than 20, samples from a nearby village were used to supplement up to 20. The collected samples were frozen a – 80 °C for at least 72 h and sandwich ELISA (Dog *Echinococcus* coproantigens ELISA kit, Combined, Shenzhen, China) was used to detect infection states of dogs.

### Data collection and analysis

Data on dog registration, management and deworming, and stray dog accommodation were collected through retrospective surveys and field study. The results of the 2016 TAR echinococcosis prevalence survey were used as a baseline data for dog infection rate. The dog infection rate in 2019 was based on data from the Central Government's Transfer Payment Project for Echinococcosis Control. The collected data were firstly systematized and checked before being entered and analyzed using SPSS 20.0 (IBM, Armonk, USA). A chi-square test was used to compare the dog infection rate in two cross-sections. Geographic information maps were mapped using ArcGIS version 10.1 (ESRI, Redlands, USA).

## Results

### Control measures for domestic dogs

#### Registration and management of domestic dogs

**Fig. 1:**
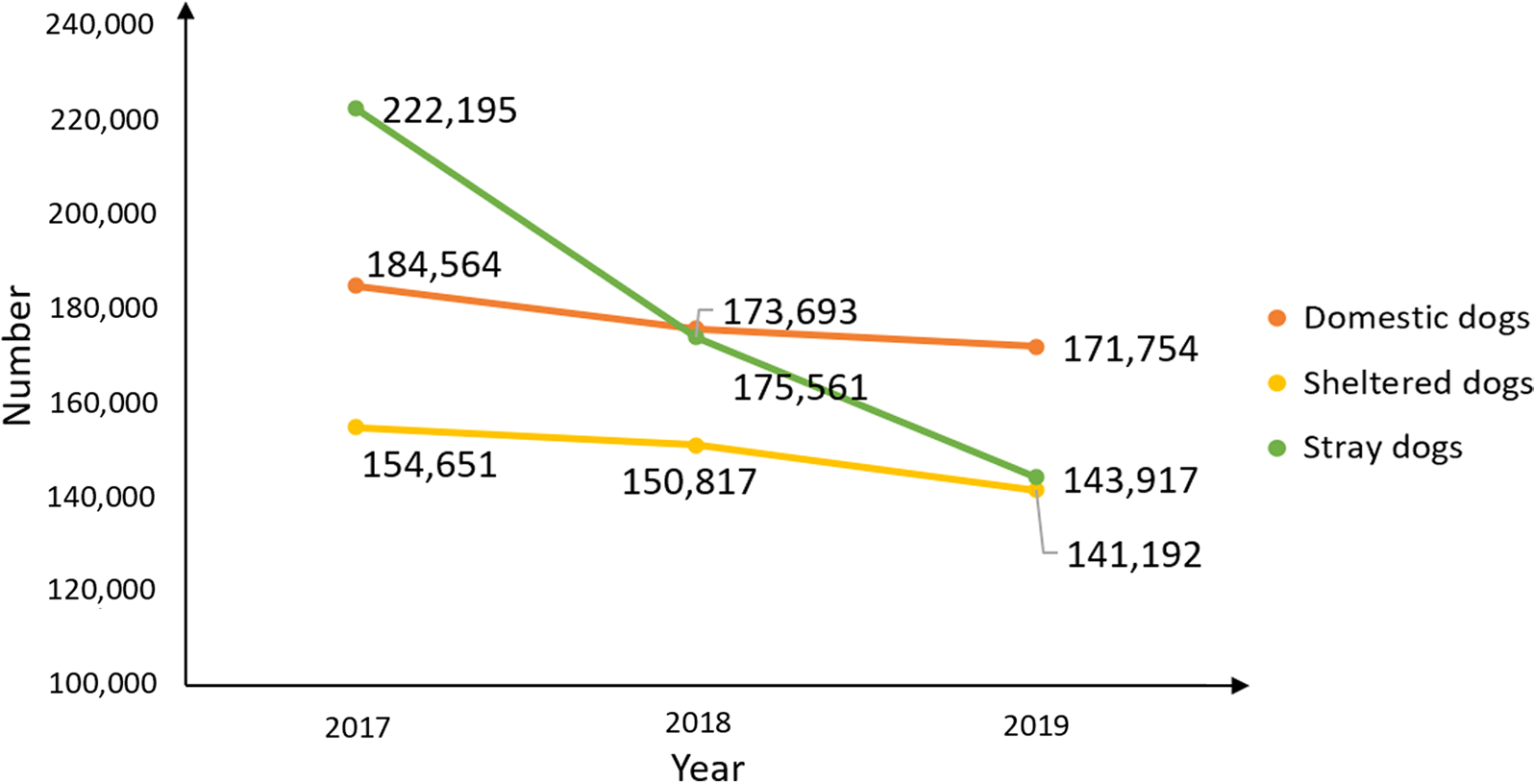
Dog populations' evolution in Tibet Autonomous Region during 2017–2019

The registration rate of domestic dogs increased annually from 78.4% in 2017 to 88.8% in 2018 and 99.0% in 2019 (Table 1 and Fig. 1). Currently, all domestic dogs in each region have been registered for management and are tethered. The number of domestic dogs decreased from 184,564 to 175,561 between 2017 and 2018, and then to 171,754 in 2019 (Table 1). The 2018 surveys of domestic dogs revealed that TAR had 670,838 households and an average of 1 dog for four households.

**Table 1:** Registration and management of domestic dogs by prefecture/city in Tibet Autonomous Region during 2017–2019

Prefecture/City		2017			2018			2019		
	Endemic county	Total domestic dogs	Registered domestic dogs	Registration rate of domestic dogs (%)	Total domestic dogs	Registered domestic dogs	Registration rate of domestic dogs (%)	Total domestic dogs	Registered domestic dogs	Registration rate of domestic dogs (%)
Lhasa		24,744	24,743	100.0	23,807	23,807	100.0	26,883	26,883	100.0
	Chengguan	7885	7885	100.0	10,091	10,091	100.0	11,734	11,734	100.0
	Linzhou	3158	3158	100.0	1327	1327	100.0	4485	4485	100.0
	Dangxiong	2193	2193	100.0	3827	3827	100.0	1186	1186	100.0
	Nimu	1776	1776	100.0	1625	1625	100.0	1535	1535	100.0
	Qushui	3130	3130	100.0	1650	1650	100.0	2895	2895	100.0
	Duilong Deqing	1205	1205	100.0	661	661	100.0	716	716	100.0
	Dazi	3892	3892	100.0	3458	3458	100.0	3219	3219	100.0
	Mozhu Gongka	1505	1504	100.0	1168	1168	100.0	1113	1113	100.0
Changdu		28,033	20,208	72.1	28,033	23,244	82.9	28,033	27,962	99.8
	Karuo	3985	2897	72.7	3985	3285	82.4	3985	3892	97.7
	Jiangda	5085	3645	71.7	5085	4146	81.5	5085	4984	98.0
	Gongjue	956	756	79.1	956	817	85.5	956	949	99.3
	Leiwuqi	846	609	72.0	846	696	82.3	846	829	98.0
	Dingqing	3528	2563	72.6	3528	2846	80.7	3528	3877	100.0
	Chaya	2236	1617	72.3	2236	1887	84.4	2236	2237	100.0
	Basu	2220	1598	72.0	2220	1846	83.2	2220	2152	96.9
	Zuogong	1800	1309	72.7	1800	1522	84.6	1800	1743	96.8
	Mangkang	4150	2914	70.2	4150	3527	85.0	4150	4088	98.5
	Luolong	1129	799	70.8	1129	946	83.8	1129	1135	100.0
	Bianba	2098	1501	71.5	2098	1726	82.3	2098	2076	99.0
Shannan		12,434	3858	31.0	12,787	9844	77.0	12,988	12,988	100.0
	Naidong	3700	988	26.7	3081	2,467	80.1	3081	3081	100.0
	Zanang	1088	540	49.6	1595	990	62.1	1599	1599	100.0
	Gongga	1941	344	17.7	2402	1606	66.9	2475	2475	100.0
	Sangri	401	184	45.9	543	412	75.9	557	557	100.0
	Qiongjie	1120	379	33.8	548	454	82.9	548	548	100.0
	Qusong	390	142	36.4	428	425	99.3	428	428	100.0
	Cuomei	264	162	61.4	307	264	86.0	308	308	100.0
	Luoza	362	354	97.8	427	419	98.1	433	433	100.0
	Jiacha	1316	295	22.4	1698	1313	77.3	1713	1713	100.0
	Longzi	538	127	23.6	593	541	91.2	680	680	100.0
	Cuona	352	98	27.8	222	197	88.7	223	223	100.0
	Langkazi	962	245	25.5	943	756	80.2	943	943	100.0
Shigatse		50,067	31,678	63.3	42,703	31,678	74.2	41,074	39,495	96.2
	Sangzhuzi	8898	3123	35.1	4542	3123	68.8	4268	3548	100.0
	Nanmulin	3373	1127	33.4	3558	3558	100.0	3360	3360	100.0
	Jiangzi	3473	499	14.4	4299	499	11.6	4299	4299	100.0
	Dingri	1957	931	47.6	6662	6662	100.0	6662	6662	100.0
	Sajia	3181	1327	41.7	3450	3450	100.0	3450	3450	100.0
	Lazi	1673	100	6.0	1747	1747	100.0	1747	1747	100.0
	Angren	1701	1008	59.3	1701	1008	59.3	1008	1008	100.0
	Xietongmen	1384	617	44.6	1617	1617	100.0	1617	1617	100.0
	Bailang	3038	1914	63.0	2823	1914	67.8	2823	2225	100.0
	Renbu	1339	1136	84.8	1472	1136	77.2	1393	1236	100.0
	Kangma	1936	1708	88.2	1708	1708	100.0	1708	1708	100.0
	Dingjie	1648	284	17.2	1116	284	25.5	1239	1239	100.0
	Zhongba	6924	1,450	20.9	1537	1,537	100.0	1537	1537	100.0
	Yadong	655	487	74.4	483	487	100.0	495	495	98.0
	Jilong	1071	488	45.6	670	448	66.9	573	481	100.0
	Nielamu	5979	1080	18.1	3400	1,080	31.8	3400	3388	100.0
	Saga	1248	750	60.1	1248	750	60.1	825	825	100.0
	Gangba	589	313	53.1	670	670	100.0	670	670	100.0
Naqu		43,629	42,767	98.0	43,364	43,183	99.6	40,590	40,590	100.0
	Naqu County	11,064	11,064	100.0	6707	6707	100.0	6157	6157	100.0
	Jiali	1032	1032	100.0	3101	3101	100.0	2962	2962	100.0
	Biru	5754	4892	85.0	6592	6592	100.0	6261	6261	100.0
	Nierong	4377	4377	100.0	4838	4838	100.0	4850	4850	100.0
	Anduo	2480	2480	100.0	2493	2493	100.0	2507	2507	100.0
	Shenza	1027	1027	100.0	1054	873	82.8	224	224	100.0
	Suoxian	3451	3451	100.0	405	3405	100.0	3410	3410	100.0
	Bange	2829	2829	100.0	2875	2875	100.0	1787	1787	100.0
	Baqing	7384	7384	100.0	6360	6360	100.0	6567	6567	100.0
	Nima	3660	3660	100.0	4616	4616	100.0	4634	4634	100.0
	Shuanghu	571	571	100.0	1323	1323	100.0	1231	1231	100.0
Ali		8250	8250	100.0	8355	8355	100.0	9523	9523	100.0
	Pulan	330	330	100.0	117	117	100.0	1,681	1681	100.0
	Zhada	323	323	100.0	234	234	100.0	278	278	100.0
	Gaer	213	213	100.0	1323	1323	100.0	1056	1056	100.0
	Ritu	1438	1438	100.0	1206	1206	100.0	953	953	100.0
	Geji	3386	3386	100.0	3363	3363	100.0	2485	2485	100.0
	Gaize	1740	1740	100.0	1495	1495	100.0	1596	1596	100.0
	Cuoqin	820	820	100.0	617	617	100.0	1474	1474	100.0
Linzhi [12]		17,407	13,216	75.9	16,512	15,766	95.5	12,663	12,491	98.6
	Linzhi County	3759	2722	72.4	2820	2679	95.0	2669	2643	99.0
	Gongbu Jiangda	3759	3759	100.0	3310	3310	100.0	2305	2259	98.0
	Milin	3287	1446	44.0	2651	2545	96.0	2168	2147	99.0
	Motuo	1193	453	38.0	1054	1011	95.9	401	397	99.0
	Bomi	2863	2863	100.0	2771	2660	96.0	2661	26,08	98.0
	Chayu	1912	1339	70.0	2126	1781	83.8	2048	2028	99.0
	Langxian	634	634	100.0	1780	1780	100.0	411	409	99.5
Total	74 counties	184,564	144,720	78.4	175,561	155,877	88.8	171,754	169,932	99.0

#### Deworming of domestic dogs

In 2017, dogs were dewormed at an average of five times a year. In 2018 and 2019, this number was increased to 12, ensuring that every dog was dewormed monthly as required by the Plan. As by December 31, 2019, there have been more than 5.09 million deworming doses for domestic dogs in the past three years (Table 2).

**Table 2:** Deworming of domestic dogs by prefecture/city in Tibet Autonomous Region during 2017–2019

Prefecture/City	2017			2018			2019		
	Total domestic dogs	Dog deworming doses	Annual average dog deworming doses	Total domestic dogs	Dog deworming doses	Annual average dog deworming doses	Total domestic dogs	Dog deworming doses	Annual average dog deworming doses
Lhasa	24,744	149,969	6	23,807	304,494	13	26,883	322,588	12
Changdu	28,033	118,354	4	28,033	353,743	13	28,033	331,566	12
Shannan	12,434	10,759	1	12,787	122,928	10	12,988	155,856	12
Shigatse	50,067	74,770	1	42,703	492,613	12	41,074	533,962	13
Naqu	43,629	377,711	9	43,364	498,437	11	40,590	489,901	12
Ali	8250	99,000	12	8355	100,260	12	9523	114,276	12
Linzhi [12]	17,407	67,352	4	16,512	204,375	12	12,663	171,315	14
Total	184,564	897,915	5	175,561	2,076,850	12	171,754	2,119,464	12

### Control measures for stray dogs

**Fig. 2:**
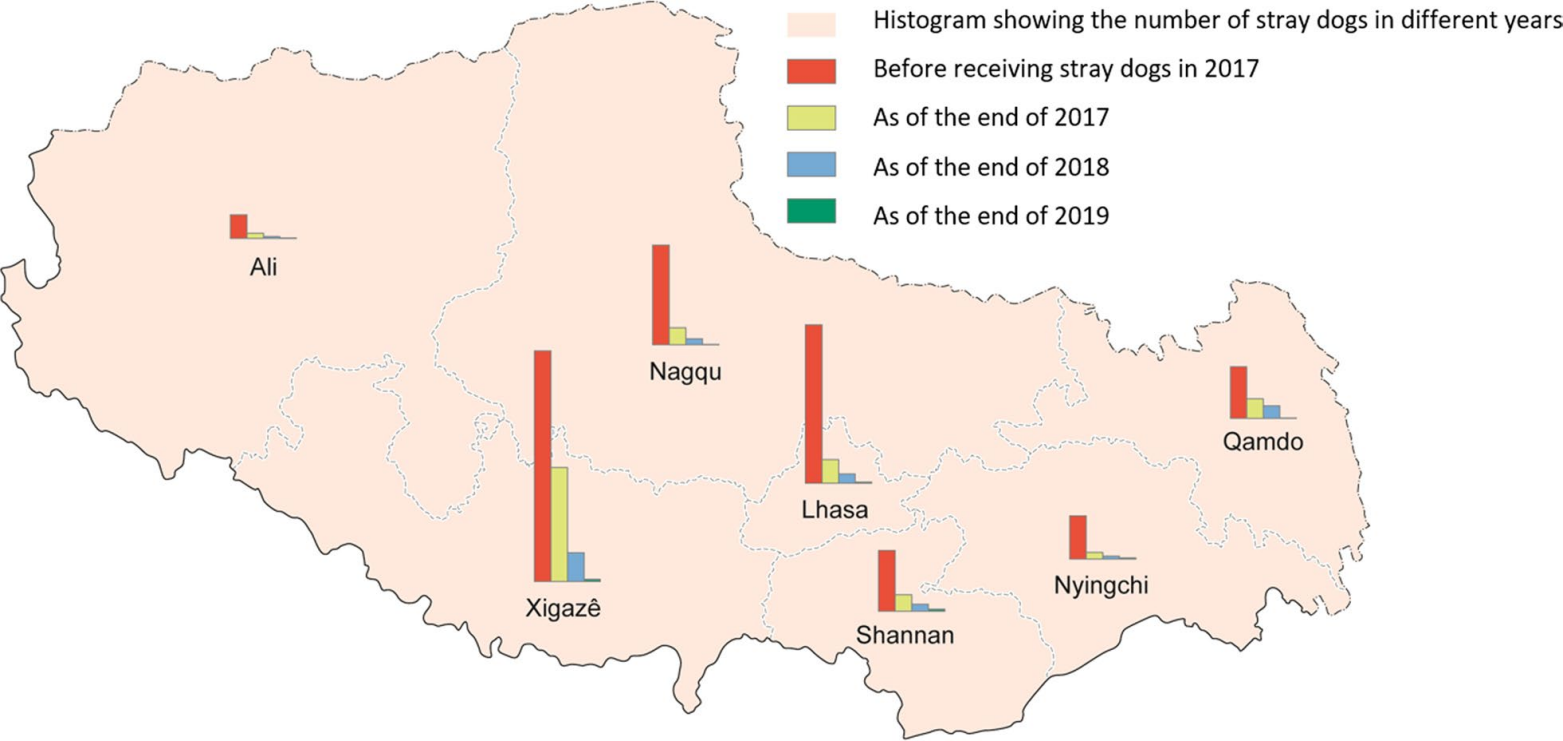
Distribution of changes in the number of stray dogs in each city (prefecture) of Tibet Autonomous Region. Map approval No. GS (2022) 2437

From 2017 to 2019, 69.6%, 86.8%, and 98.1% of stray dogs were sheltered, respectively (Table 3). The sharp reduction of the number of stray dogs (Figs. 1 and 2), resulted in an overall reduction of the number of infection sources. Assessment teams reported that stray dog sheltering management made remarkable achievements, and stray dogs were rarely observed in endemic villages. No dog feces were found on the roadside or around settlements during the visits.

**Table 3:** Stray dog sheltering by prefecture/city in Tibet Autonomous Region during 2017–2019

Prefecture/City	2017				2018				2019			
	No. of stray dogs	No. of sheltered dogs	No. of stray dogs at end of year	Sheltering rate (%)	No. of stray dogs	No. of sheltered dogs	No. of stray dogs at end of year	Sheltering rate (%)	No. of stray dogs	No. of sheltered dogs	No. of stray dogs at end of year	Sheltering rate (%)
Lhasa	52,571	44,811	7760	85.2	30,764	27,727	3037	90.1	24,995	24,659	336	98.7
Changdu	17,200	10,706	6494	62.2	16,081	11,820	4261	73.5	16,420	16,256	164	99.0
Shannan	20,254	14,676	5578	72.5	14,324	12,004	2320	83.8	9370	8612	758	91.9
Shigatse	76,687	38,651	38,036	50.4	71,569	72,649	9617	86.6	58,822	58,040	782	98.7
Naqu	33,234	27,474	5760	82.7	22,208	20,228	1980	91.1	18,536	18,383	153	99.2
Ali	7913	6201	1712	78.4	5680	5023	657	88.4	3937	3804	133	96.6
Linzhi [12]	14,336	12,132	2204	84.6	13,067	12,063	1004	92.3	11,837	11,438	399	96.6
Total	222,195	154,651	67,544	69.6	173,693	150,817	22,876	86.8	143,917	141,192	2725	98.1

### Status of dogs as sources of infection

**Fig. 3:**
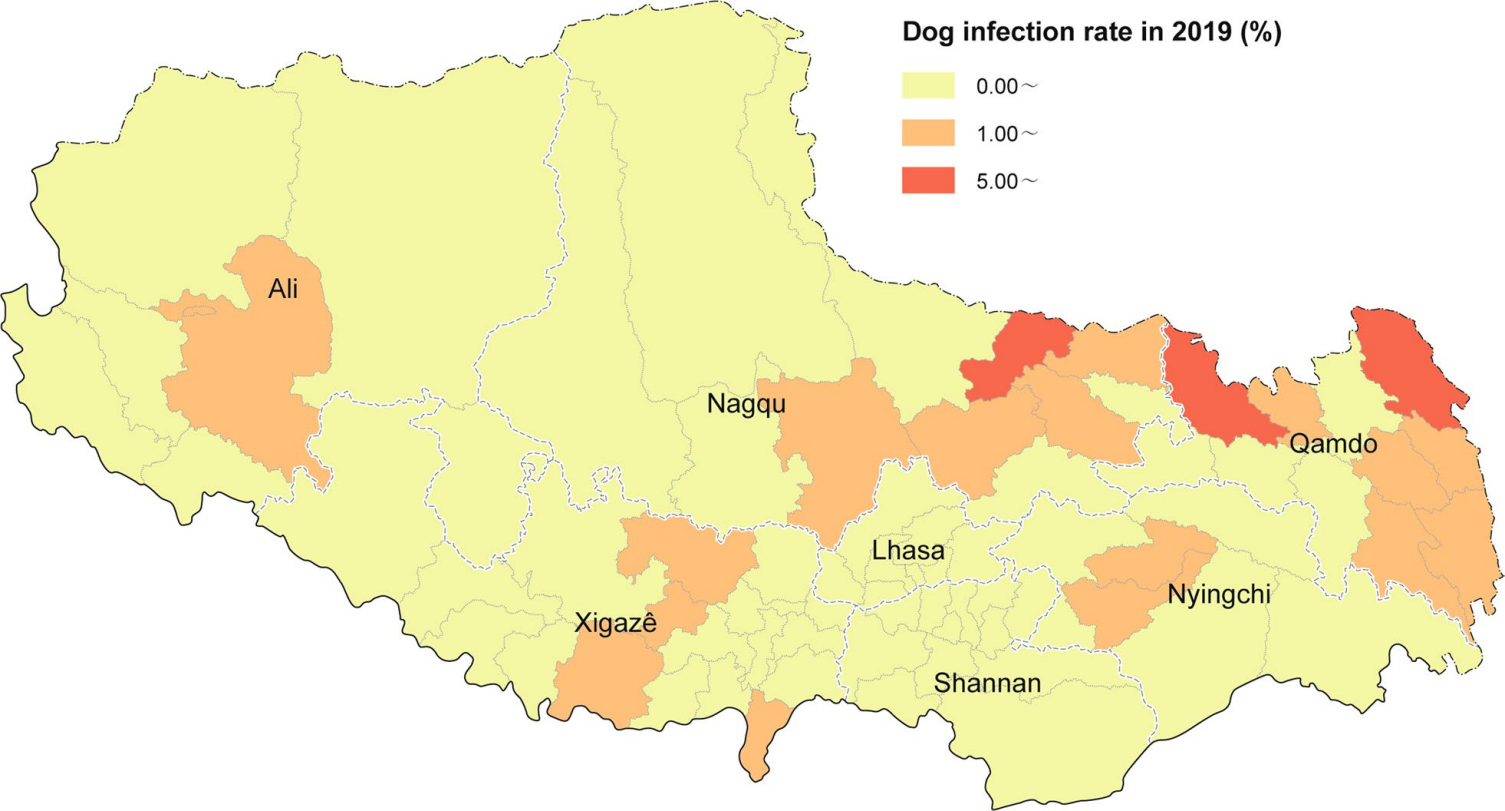
Dog infection distribution of comprehensive control effect of echinococcosis in Tibet Autonomous Region in 2019. Map approval No. GS (2022) 2437

The assessments revealed that in 2019 the dog infection rate in TAR was 1.7% (252/14,584). At present, out of 74 endemic counties in TAR, 3 reported a dog infection rate higher than 5%, 16 reported a rate of between 1 and 5%, and 55 reported a rate of < 1%, including 32 counties with no reported positive dog feces (Table 4) (Fig. 3).

**Table 4:** Comparison of domestic dog infections in prefecture/cities of Tibet Autonomous Region in 2016 vs 2019

Prefecture/City	2016			2019			Test result		
	No. of dog feces tested	No. of dog feces positive	Positive rate of dog feces (%) [95% *CI*]	No. of dog feces tested	No. of dog feces positive	Positive rate of dog feces (%) [95% *CI*]	*χ*^2^	*P*	Result
Lhasa	1047	66	6.3 [4.8, 7.8]	2,620	0	0.0	168.19	< 0.01	↓
Changdu	1358	78	5.74 [4.5, 7.0]	1,669	56	3.4 [2.5, 4.2]	10.10	< 0.01	↓
Shannan	1046	107	10.2 [8.4, 12.1]	1,741	0	0.0	185.21	< 0.01	↓
Shigatse	1945	94	4.8 [3.9, 5.8]	1,803	41	2.3 [1.6, 3.0]	17.65	< 0.01	↓
Naqu	1127	128	11.4 [9.5, 13.2]	2,941	67	2.3 [1.7, 2.8]	147.18	< 0.01	↓
Ali	423	34	8.04 [5.4, 10.6]	1,095	11	1.0 [0.4, 1.6]	52.47	< 0.01	↓
Linzhi [12]	618	45	7.3 [5.2, 9.3]	2,715	77	2.8 [2.2, 3.5]	28.21	< 0.01	↓
Total	7564	552	7.3 [6.7, 7.9]	14,584	252	1.7 [1.5, 1.9]	441.68	< 0.01	↓

This table represents changes in the infection rate of domestic dogs. Due to the large number of stray dogs sheltered and effective management in endemic counties, the number of infection sources has decreased significantly, and the weighted dog infection rate, which represents the prevalence of endemic counties, has been significantly reduced. *CI* Confidence interval

### Assessment of the effect of infection source control

**Fig. 4:**
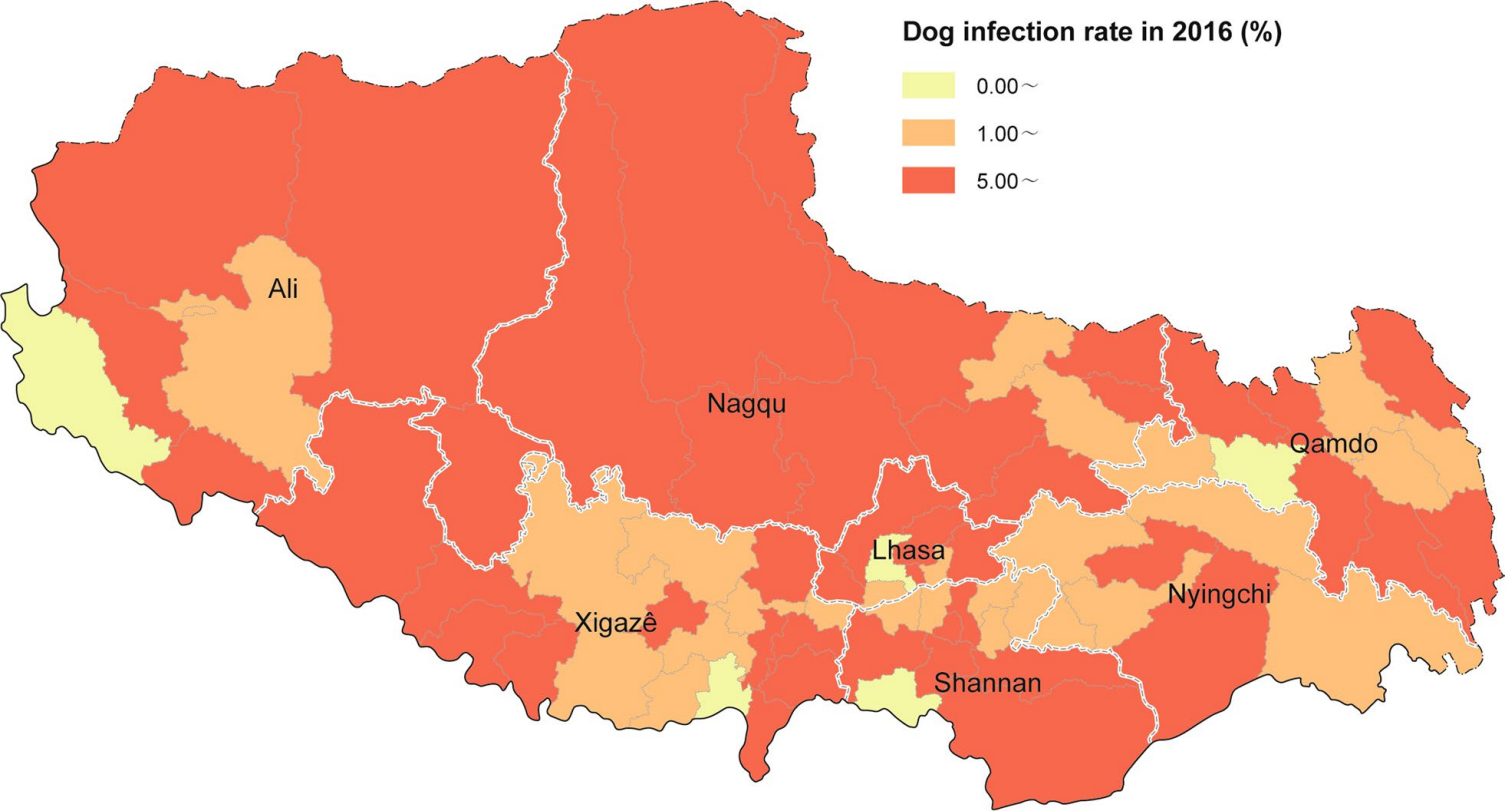
Distribution of dog infections in echinococcosis prevalence surveys in Tibet Autonomous Region in 2016. Map approval No. GS (2022) 2437

In 2016, 43 Class I counties were identified with a dog infection rate ≥ 5% (Fig. 4), 26 Class II counties with a dog infection rate of < 5% and ≥ 1%, and 5 Class III counties with a dog infection rate of < 1%. In 2019, the numbers of Class I, II, and III counties were 3, 16, and 55, respectively (Fig. 4).

**Fig. 5:**
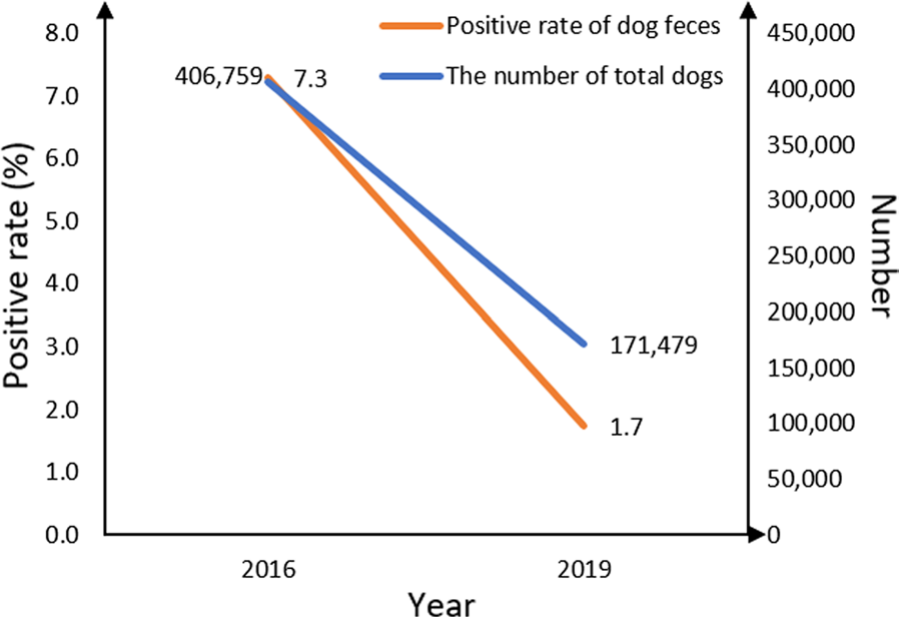
The number of dogs and positive rate of dogs with *Echinococcus* spp. in Tibet Autonomous Region

The dog infection rate was 1.7% (252/14,584) in 2019, significantly lower (*P* < 0.05) than in 2016 when it was 7.3%, representing a 76.3% decrease. County-level dog infection rates ranged from 41.3% in 2016 (Baqing County) to 6.2% in 2019. The dog infection rate of the seven cities or prefectures under the jurisdiction of TAR all declined significantly (*P* < 0.05), as shown in Table 4. Given the important role of decreased infection sources in lowering the risk of local transmission, the dog infection rate in 2019 was weighed and compared to that of 2016. The number of dogs were different in the same endemic county in 2016 and 2019. Therefore, the positive rates of dog were weighted adjusted based on the number of domestic dogs in 2016 and 2019. The results are presented in Table 5 and Fig. 5. The weighted overall infection rate in 2019 was 0.7%, with a decrease of 90.0% compared to that of 2016 (Fig. 5). Rates were compared for each endemic county, and each county experienced a reduction in the rate to varying degrees. The decrease was significant in 32 counties (*P* < 0.05) and insignificant in 40 counties (*P* > 0.05). The dog infection rate has always been null in two counties, Zanda County of Ali Prefecture and Lhozhag County of Shannan City.

**Table 5:** Comparison of dog infections in 74 endemic counties of Tibet Autonomous Region in 2016 vs 2019

Region/Prefecture/League/City	Endemic county	2016				2019				Weighted adjusted positive rate	Test results of two rates
		Total dogs	No. of dog feces tested	No. of dog feces positive	Positive rate of dog feces (%) [95% *CI*]	Total dogs	No. of dog feces tested	No. of dog feces positive	Positive rate of dog feces (%) [95% *CI*]		
Lhasa	Chengguan	25,186	324	29	9.0 [5.8, 12.1]	11,754	1060	0	0.0	0.0	*P* < 0.05
	Linzhou	10,664	159	15	9.4 [4.8, 14.0]	4536	100	0	0.0	0.0	*P* < 0.05
	Dangxiong	6209	80	6	7.5 [1.6, 13.4]	1239	500	0	0.0	0.0	*P* < 0.05
	Nimu	4562	80	4	5.0 [0.1, 9.9]	1568	80	0	0.0	0.0	*P* > 0.05
	Qushui	8146	80	2	2.5 [− 1.0, 6.0]	2922	200	0	0.0	0.0	*P* > 0.05
	Duilong Deqing	8221	163	1	0.6 [− 0.6, 1.8]	757	100	0	0.0	0.0	*P* > 0.05
	Dazi	6738	80	3	3.8 [− 0.5, 8.0]	3258	300	0	0.0	0.0	*P* > 0.05
	Mozhu Gongka	7589	81	6	7.4 [1.6, 13.2]	1185	280	0	0.0	0.0	*P* < 0.05
Changdu	Karuo	5928	319	5	1.6 [0.2, 2.9]	4008	120	0	0.0	0.0	*P* > 0.05
	Jiangda^a^	7468	160	8	5.0 [1.6, 8.4]	5105	108	10	9.3 [3.7, 14.8]	6.3	*P* > 0.05
	Gongjue	1910	80	2	2.5 [− 1.0, 6.0]	972	100	3	3.0 [− 0.4, 6.4]	1.5	*P* > 0.05
	Leiwuqi^a^	1406	80	7	8.8 [2.4, 15.1]	856	100	4	4.0 [0.1, 7.9]	2.4	*P* > 0.05
	Dingqing^a^	3972	160	11	6.9 [2.9, 10.8]	3,548	280	18	6.4 [3.5, 9.3]	5.7	*P* > 0.05
	Chaya	4575	80	2	2.5 [− 1.0,6.0]	2246	100	7	7.0 [1.9, 12.1]	3.4	*P* > 0.05
	Basu	4484	79	6	7.6 [1.6, 13.6]	2230	280	3	1.1 [− 0.1, 2.3]	0.5	*P* < 0.05
	Zuogong^a^	3476	80	20	25.0 [15.3, 34.7]	1820	101	4	4.0 [0.1, 7.8]	2.1	*P* < 0.05
	Mangkang	5654	160	15	9.4 [4.8, 13.9]	4168	280	5	1.8 [0.2, 3.3]	1.3	*P* < 0.05
	Luolong	3997	80	0	0.0	1139	100	2	2.0 [− 0.8, 4.8]	0.6	*P* > 0.05
	Bianba	2363	80	2	2.5 [− 1.0, 6.0]	2105	100	0	0.0	0.0	*P* > 0.05
Shannan	Naidong	7195	140	9	6.4 [2.3, 10.5]	3149	101	0	0.0	0.0	*P* < 0.05
	Zanang	3034	80	3	3.8 [− 0.5, 8.0]	1641	100	0	0.0	0.0	*P* > 0.05
	Gongga	4422	80	3	3.8 [− 0.5, 8.0]	2548	100	0	0.0	0.0	*P* > 0.05
	Sangri	1457	72	3	4.2 [− 0.6, 8.9]	590	100	0	0.0	0.0	*P* > 0.05
	Qiongjie	2666	80	4	5.0 [0.1, 9.9]	589	100	0	0.0	0.0	*P* > 0.05
	Qusong	876	80	1	1.3 [− 1.2, 3.7]	476	280	0	0.0	0.0	*P* > 0.05
	Cuomei	1240	114	43	37.7 [28.7, 46.8]	365	100	0	0.0	0.0	*P* < 0.05
	Luoza^b^	2226	80	0	0.0	547	100	0	0.0	0.0	/
	Jiacha	2534	80	2	2.5 [− 1.0, 6.0]	1783	280	0	0.0	0.0	*P* > 0.05
	Longzi	2646	80	6	7.5 [1.6, 13.4]	763	280	0	0.0	0.0	*P* < 0.05
	Cuona	902	80	11	13.8 [6.0, 21.5]	259	100	0	0.0	0.0	*P* < 0.05
	Langkazi	3490	80	22	27.5 [17.5, 37.5]	1036	100	0	0.0	0.0	*P* < 0.05
Shigatse	Sangzhuzi	20,679	320	8	2.5 [0.8, 4.2]	4308	100	0	0.0	0.0	*P* > 0.05
	Nanmulin	12,132	160	13	8.1 [3.8, 12.4]	415	100	0	0.0	0.0	*P* < 0.05
	Jiangzi	11,312	160	8	5.0 [1.6, 8.4]	4334	100	0	0.0	0.0	*P* > 0.05
	Dingri	6081	154	4	2.6 [0.1, 5.1]	6706	101	1	1.0 [− 1.0, 3.0]	1.1	*P* > 0.05
	Sajia	4445	80	3	3.8 [− 0.5, 8.0]	3489	100	0	0.0	0.0	*P* > 0.05
	Lazi	4805	80	5	6.3 [0.8, 11.7]	1802	101	8	7.9 [2.6, 13.3]	3.0	*P* > 0.05
	Angren	11,470	160	6	3.8 [0.8, 6.7]	1053	100	3	3.0 [− 0.4, 6.4]	0.3	*P* > 0.05
	Xietongmen	7168	80	3	3.8 [− 0.5, 8.0]	1662	100	7	7.0 [1.9, 12.1]	1.6	*P* > 0.05
	Bailang	5954	80	5	6.3 [0.8, 11.7]	2868	101	2	2.0 [0.8, 4.7]	1.0	*P* > 0.05
	Renbu	2154	83	1	1.2 [− 1.2, 3.6]	1443	100	1	1.0 [− 1.0, 3.0]	0.7	*P* > 0.05
	Kangma	5060	80	6	7.5 [1.6, 13.4]	1748	100	0	0.0	0.0	*P* < 0.05
	Dingjie	3327	80	2	2.5 [− 1.0, 6.0]	1279	100	2	2.0 [− 0.8, 4.8]	0.8	*P* > 0.05
	Zhongba	8597	80	8	10.0 [3.3, 16.7]	1587	100	3	3.0 [− 0.4, 6.4]	0.6	*P* < 0.05
	Yadong	2551	28	5	17.9 [2.7, 33.0]	530	100	8	8.0 [2.6, 13.4]	1.7	*P* < 0.05
	Jilong	2836	80	6	7.5 [1.6, 13.4]	613	100	1	1.0 [− 1.0, 3.0]	0.2	*P* < 0.05
	Nielamu	11,162	80	5	6.3 [0.8, 11.7]	3445	100	1	1.0 [− 1.0, 3.0]	0.3	*P* < 0.05
	Saga	4445	80	6	7.5 [1.6, 13.4]	863	100	1	1.0 [− 1.0, 3.0]	0.2	*P* < 0.05
	Gangba	2576	80	0	0.0	711	100	3	3.0 [− 0.4, 6.4]	0.8	*P* > 0.05
Naqu City	Naqu County	15,011	322	23	7.1 [4.3, 10.0]	6170	101	5	5.0 [0.6, 9.3]	2.0	*P* > 0.05
	Jiali	2979	80	10	12.5 [5.1, 19.9]	2999	950	0	0.0	0.0	*P* < 0.05
	Biru	9927	160	4	2.5 [0.1, 4.9]	6275	100	6	6.0 [1.3, 10.7]	3.8	*P* > 0.05
	Nierong	7500	80	3	3.8 [− 0.5, 8.0]	4873	214	25	11.7 [7.3, 16.0]	7.6	*P* > 0.05
	Anduo	4015	79	10	12.7 [5.2, 20.2]	2515	500	4	0.8 [0, 1.6]	0.5	*P* < 0.05
	Shenza	1900	80	11	13.8 [6.0, 21.5]	237	100	0	0.0	0.0	*P* < 0.05
	Suoxian	11,138	80	4	5.0 [0.1, 9.9]	3423	100	0	0.0	0.0	*P* > 0.05
	Bange	4737	80	14	17.5 [9.0, 26.0]	1793	220	10	4.6 [1.8, 7.3]	1.7	*P* < 0.05
	Baqing	12,530	46	19	41.3 [26.5, 56.1]	6578	276	17	6.2 [3.3, 9.0]	3.2	*P* < 0.05
	Nima	5196	80	16	20.0 [11.0, 29.0]	4640	100	0	0.0	0.0	*P* < 0.05
	Shuanghu^a^	1930	40	14	35.0 [19.6, 50.4]	1240	280	0	0.0	0.0	*P* < 0.05
Ali	Pulan	1188	40	4	10.0 [0.3, 19.7]	1696	100	0	0.0	0.0	*P* < 0.05
	Zhada^b^	550	25	0	0.0	288	100	0	0.0	0.0	/
	Gaer	1165	80	6	7.5 [1.6, 13.4]	1076	200	1	0.5 [− 0.5, 1.5]	0.5	*P* < 0.05
	Ritu	1897	40	2	5.0 [− 2.1, 12.1]	962	180	1	0.6 [− 0.5, 1.7]	0.3	*P* > 0.05
	Geji	5266	79	2	2.5 [− 1.0, 6.1]	2510	115	3	2.6 [− 0.3, 5.6]	1.2	*P* > 0.05
	Gaize	4539	79	15	19.0 [10.1, 27.8]	1632	200	5	2.5 [0.3, 4.7]	0.9	*P* < 0.05
	Cuoqin	1558	80	5	6.3 [0.8, 11.7]	1492	200	1	0.5 [− 0.5, 1.5]	0.5	*P* < 0.05
Linzhi City [12]	Linzhi County^a^	6775	117	27	23.1 [15.3, 30.8]	2783	1, 058	40	3.8 [2.6, 4.9]	1.6	*P* < 0.05
	Gongbu Jiangda	6673	81	4	4.9 [0.1, 9.8]	2393	166	2	1.2 [− 0.5, 2.9]	0.4	*P* > 0.05
	Milin	5351	80	1	1.3 [− 1.2, 3.7]	2229	192	5	2.6 [0.3, 4.9]	1.1	*P* > 0.05
	Motuo	2988	80	6	7.5 [1.6, 13.4]	432	120	2	1.7 [− 0.7, 4.0]	0.2	*P* < 0.05
	Bomi	5578	80	1	1.3 [− 1.2, 3.7]	2721	205	2	1.0 [− 0.4, 2.3]	0.5	*P* > 0.05
	Chayu^a^	2898	100	3	3.0 [− 0.4, 6.4]	2078	200	0	0.0	0.0	*P* > 0.05
	Langxian	1480	80	3	3.8 [− 0.5, 8.0]	426	774	26	3.4 [2.1, 4.6]	1.0	*P* > 0.05
Total	74 counties	406,759	7,564	552	7.3 [6.7, 7.9]	171, 479	14, 584	252	1.7 [1.5, 1.9]	0.7	*P* < 0.05

In the process of testing the two rates, different methods were selected according to data characteristics, and Fisher's exact test was used in most cases

*CI* Confidence interval

^a^Chi-square test

^b^Both rates were 0, and therefore no test was performed

## Discussion

China displays the highest echinococcosis prevalence worldwide, and TAR displays the highest echinococcosis prevalence in China [4, 5]. Echinococcosis has developed into a major public health issue, severely impeding economic development, ethnic unity, and social stability of TAR and endangering health and safety of people. TAR has a large pastoral area. Animal husbandry is the main way of production and life for local residents. Tibetan herdsmen families have a traditional habit of keeping dogs to protect their livestock, while pastoralists and Buddhist monks are easily tolerating stray dogs. These habits have been linked to an increased risk of human echinococcosis [13]. Experimental studies have demonstrated that dogs can keep an independent *E. multilocularis* transmission cycle [6]. There is evidence confirming the hypothesis that wild dogs serve as a reservoir of *E. granulosus* transmission due to transmission between wild and domestic hosts [14, 15]. While infection rates would decline if dogs cannot wander freely [16], dogs still represent the greatest risk to people [17–19]. Therefore, it is crucial to reduce the incidence of human parasitic infection rates by implementing control measures in animal hosts, namely dogs in this case [20]. Feasible intervention measures of wild hosts and domestic dogs are critical for reducing transmission risks of *E. granulosus* and *E*. *multilocularis* [21]. Control measures for *E. granulosus* are theoretically more governable in domestic animals [22]. Generally, humans infected with echinococcosis do not cause active transmission of Cystic echinococcosis unless a dog ingests hydatid cysts [23]. Deworming dogs is an effective measure to quickly reduce active transmission [23]. In addition, the density of dog feces is higher around villages with frequent human activities [19]. The positive rate of *E. granulosus* antigen in dog feces was not related to the density of livestock within its range, but the positive rate of fecal *E*. *multilocularis* antigen of domestic dogs was positively correlated with the number of stray dogs visible within 200 m of the activity diameter of domestic dogs [24, 25].

The central finance transfer payment local echinococcosis control project has been launched in 2005. Since 2008, TAR has been included in two counties to carry out epidemiological investigation. Due to the limitations of local conditions, other counties have carried out epidemiological investigation one after another, but the process was slow. In response to the severe situation of echinococcosis prevalence, TAR established a comprehensive echinococcosis control headquarters in February 2017 to coordinate and implement various control measures of stray dogs, limit domestic dogs, and communicate the need to reduce the number of untethered dogs and keep dogs. These measures included population investigation and treatment, domestic dog registration and deworming, stray dog sheltering, livestock immunization, and quality of drinking water. Specifically, the government enforced the control of infection sources by limiting the number of dogs, registering them, and issuing domestic dog certificates via an electronic registration system. Stray dogs were captured and sent to the nearest shelters for management. The population of domestic dogs did not decline significantly but remained relatively stable, implying that public awareness of stray dog sheltering and tethering led to an absence of increase. Before 2016, TAR had a substantially large number of dogs, with almost every family owing at least one dog. However, although no statistics on dog breeding are available. As echinococcosis control programs, health education, and people awareness advanced, people have become aware of animal hazards, have reduced the number of dogs in their families, or have even completely stopped feeding dogs. After three years of control practices, the number of stray dogs was effectively reduced. Up to now, TAR has spent CNY 37 million on building 84 stray dog shelters, and 446,660 stray or infected dogs were captured, sheltered or euthanized. The drastic reduction in stray dogs played a vital role in controlling infection sources and significantly reducing the risk of echinococcosis transmission. The dog infection rate has always been a sensitive indicator of the local prevalence and risk of cystic echinococcosis transmission.

To ensure that every dog is dewormed monthly, government departments supervised the deworming practices of domestic dogs in each village. Simultaneously, a multi-dimensional, multi-index assessment and evaluation system for echinococcosis control measures was established. In TAR, the number of stray dogs has decreased sharply, and domestic dogs have been tethered and incorporated into standardized management. On-site sampling surveys confirmed that dog deworming drugs were properly distributed. Although most dogs have achieved deworming monthly, the infection rate of dogs was still very high. Contradictions in dog feces test results indicate the presence of loopholes in the deworming process. This may result from false records in some areas or improperly implemented deworming measures (the dog did not swallow praziquantel, or the tablets were not mashed, and dogs ate the zanba and spit out the tablets). Low infection rates are also associated with sensitivity, specificity, and cross-reactivity of the test kits. A study estimated that the minimal burden of worms for assessing sensitivity might be 500, and had a suggestion for improving the sensitivity of the test kits that using parallel detection with two different kits at the same time or multiple sampling from one dog [26]. In a sample survey in TAR 93.4% of the villagers expressed their willingness to cooperate with free deworming for dogs [10]. These strategic measures are well-suited to the situation of lack of local professionals. They significantly reduce the risk of echinococcosis in the environment by focusing on the top concern, i.e., controlling the source of infection. Additionally, it provides a successful experience for other endemic counties in TAR. The measures are consistent with national control strategies and international experience with echinococcosis control [27].

The quasi eradication of stray dogs and the drastic reduction in the infection rate with *Echinococcus* spp. of domestic dogs seems to result from the TAR's efforts to control dogs as an infection source. Seven dog-targeted control programs were successfully implemented in islands states/nations, resulting in parasite elimination [28]. Iceland, New Zealand, Falkland Islands, or Tasmania have successfully eliminated CE from dogs and livestock by undertaking dog-targeted, including culling, purgation and/or anthelmintic treatments and control measures, and improving husbandry and slaughter practices [29]. However, the island is a limited geographical area, which is easier to achieve than the mainland. Additionally, some countries in South America, Europe, and East Africa have experienced success [22]. Dogs deworming successfully reduced the prevalence of *E. multilocularis* in commensal vole populations in Alaska, confirming that taking measures to protect owned dogs can reduce the risk of zoonotic transmission [30]. Iceland once released a national law stating that the effects of controlling dogs were achieved through taxation and forceful deworming, and it has been in effect since 1890 [23]. The main target of control is the definitive hosts dogs, and the aim is to reduce or eliminate the adult worm burden, which will reduce the transmission to livestock with the greatest and quickest effect [23]. TAR is expected to maintain the current mode and trend of infection source control, identify any deficiencies in control practices, strengthen supervision and quality control, improve the implementation of the computational data system with the objective of eliminating echinococcosis.

This study only assessed the management and control measures of dogs, and did not evaluate the control measures of intermediate hosts. At the same time, there was no assessment of health education in the population. In addition, the fecal sample size of some counties was a little less, and only a few villages were collected, which was difficult to represent the results of a county. Moreover, it was only tested once a year, indicating that the transmission risk in the environment had some limitations.

## Conclusions

TAR now has taken comprehensive measures to control the number of domestic and stray dogs, and achieved good results. The management and control of infectious source dogs can reduce the rate of dogs by dog registration and management, dog deworming, disposal of dog feces after deworming, reduction of dog populations, monitoring of dog infection. Comprehensive measures to control infectious source dogs were feasible and effective, and needed continuous implementation.

## Data Availability

The relevant materials and data in this study are inaccessible to peers.

## Abbreviations

AE: Alveolar echinococcosis
CE: Cystic echinococcosis
TAR: Tibet Autonomous Region
DALYs: Disability-adjusted life years
TAR CDC: Tibet Autonomous Region
NIPD: National Institute of Parasitic Diseases
China CDC: Chinese Center for Disease Control and Prevention
